# Role of C-Reactive Protein, White Blood Cell Counts, Bilirubin Levels, and Imaging in the Diagnosis of Acute Appendicitis as a Cause of Right Iliac Fossa Pain

**DOI:** 10.7759/cureus.2070

**Published:** 2018-01-15

**Authors:** Shetty Sushruth, Chellappa Vijayakumar, Krishnamachari Srinivasan, Nagarajan Raj Kumar, Gopal Balasubramaniyan, Surendra K Verma, A Ramesh

**Affiliations:** 1 Surgery, Jawaharlal Institute of Postgraduate Medical Education and Research (JIPMER), Puducherry, India.; 2 Pathology, Jawaharlal Institute of Postgraduate Medical Education and Research (JIPMER), Puducherry, India.; 3 Radiology, Jawaharlal Institute of Postgraduate Medical Education and Research (JIPMER), Puducherry, India.

**Keywords:** acute appendicitis, bilirubin level, c reactive protein, differential counts, right iliac fossa pain

## Abstract

Background

Right iliac fossa (RIF) pain is one of the most common modalities of presentation to surgical emergency. It remains a challenge to the treating clinicians to accurately diagnose or to rule out appendicitis.

Objective

The aim of the study was to compare the efficacy of clinical impression, biochemical markers, and imaging in the diagnosis of RIF pain with special reference to appendicitis and their implication in reducing the negative appendicectomy rates.

Methods

All patients presenting to casualty with RIF pain were included in the study. Blood investigations including C-reactive protein (CRP), serum bilirubin, white blood cell counts (WBC), and ultrasound (USG) were done. Based on the clinical impression, patients were either posted for appendicectomy or observed in equivocal cases. Patients who had recurrent pain on follow-up underwent appendicectomy or underwent contrast-enhanced computed tomography (CECT) in equivocal cases. Patients who only had a single self-limiting episode with no other alternative diagnosis or had a normal CECT report were included in a non-specific RIF pain group.

Results

The negative appendicectomy rate was 8.2%. The mean value of WBC counts (9.57x109/L vs 7.88x109/L; p<0.05) and that of serum bilirubin (1.37 mg/dl vs 0.89mg/dl; p<0.05) in the appendicitis and non-appendicitis group, respectively, were statistically significant. The percentage of CRP positivity was higher in the appendicitis group (51.9% vs 15%; p<0.05). The sensitivity, specificity, positive predictive value (PPV), and negative predictive value (NPV) for USG (84.2%, 77.17%, 85.4%, and 75.5%), for CRP (51.8%, 85%, 82%, and 57%), for WBC count (45.1%, 88%, 86.6%, and 48.3%), and for serum bilirubin (69.2%, 75%, 81.4%, and 60.5%) were statistically significant between the groups.

Conclusion

Imaging and biochemical investigations including bilirubin can act as useful adjuncts to the clinical diagnosis of appendicitis.

## Introduction

A wide range of laboratory investigations, scoring systems, and imaging techniques are available as an adjunct in the diagnosis for the cause of right iliac fossa (RIF) pain. White blood cell (WBC) counts and C-reactive protein (CRP) levels are commonly used in the assessment of suspected appendicitis, but their sensitivity and specificity vary widely between different studies. Some studies have shown that hyperbilirubinaemia is a useful predictor for appendicular perforation. Ultrasound (USG) has traditionally been used as an adjunct in the diagnosis of appendicitis with variable efficacy [1]. Contrast enhanced computed tomography (CECT) scan is significantly more expensive and has an added disadvantage of radiation exposure [2]. The rate of negative appendicectomies varies from 10-15% in different studies [3]. We intend to discuss ways to optimise the use of clinical findings, investigations, and imaging in the diagnosis of the cause of RIF pain with special reference to acute appendicitis.

## Materials and methods

The setting was the department of surgery of a tertiary health care centre in South India. The main objectives were to compare the levels of CRP, total and differential WBC counts, serum bilirubin, and the role of USG and CECT in various aetiologies of RIF pain with special reference to appendicitis.

All patients who presented to the casualty with complaints of RIF pain and were referred to the surgery team with age >13 years were studied. Patients who were pregnant, patients with RIF mass/abscess at presentation, patients with history of previous appendicectomy were excluded from this study. Informed consent was obtained from the patients. Detailed history and per abdomen findings were noted. All patients underwent a routine USG. Acute appendicitis was diagnosed on USG when the diameter of the appendix was >6 mm and the appendix was tender and incompressible, associated with hypertrophy of the periappendicular fat. Blood samples were collected from all patients for CRP levels, serum bilirubin, total and differential counts. The Alvarado score was calculated using 10 parameters.

Patients with definitive diagnosis of appendicitis were posted for emergency appendicectomy and postoperative histopathology reports were followed up. Patients with alternative diagnosis based on clinical or USG abdomen findings were treated accordingly or referred to relevant specialities. Patients with equivocal diagnosis were observed and discharged once their pain resolved. Patients with RIF mass formation were managed conservatively and called for interval appendicectomy.

Patients who came with recurrent pain were assessed and subjected to appendicectomy if they had signs suggestive of acute appendicitis. If the patients had an equivocal diagnosis again, they were subjected to CECT scan and posted for appendicectomy if CECT was suggestive of appendicitis. Patients with an alternative diagnosis based on CECT were managed accordingly. Patients with normal findings on CECT were reassured and kept on follow-up. All patients who were not operated and did not have a definitive diagnosis were followed up. In the patients who were symptomatic, CECT was done and managed accordingly.

Postoperative histology reports were classified as having acute uncomplicated appendicitis (simple/resolving), complicated appendicitis (perforated/gangrenous/chronic appendicitis) or as histologically normal appendix.

During the final analysis, the patients were divided into two groups. Group one (appendicitis group) included all proven cases of appendicitis by histology. Group two (non-appendicitis group) included all the patients with an alternative cause for RIF pain, the patients with histologically normal appendix, the patients who had a single self-limiting episode of RIF pain and no pain on follow-up, and the patients with recurrent pain with a normal CECT abdomen report. Twelve patients were excluded during the final analysis—seven patients with equivocal diagnosis and five patients with resolved appendicitis.

The clinical features, biochemical parameters, and USG findings were compared between the two groups. The correlation between patients with simple appendicitis and complicated appendicitis with respect to their clinical features and biochemical parameters were also analysed.

SPSS (version 19) (IBM Corp, Armonk, NY) was used to analyse the statistical data. Categorical variables were compared using Pearson’s Chi-square test and Fisher's exact probability test. The Mann-Whitney U test was used for ordinal data. The sensitivity, specificity, positive predictive value (PPV), and negative predictive value (NPV) were calculated for individual investigation. A p value of <0.05 was considered significant for all tests.

## Results

A total of 250 patients with RIF pain were included, of which 135 (54%) were males and 115 (46%) were females. Among these, 137 patients were diagnosed as appendicitis and 135 patients underwent emergency appendicectomy. The remaining two patients were found to have an alternative diagnosis intraoperatively of ileocaecal tuberculosis and ileal perforation, respectively. Out of the remaining 113 patients with RIF pain, 41 patients had an alternative diagnosis other than appendicitis and in 72 patients the diagnosis was equivocal.

**Figure 1:**
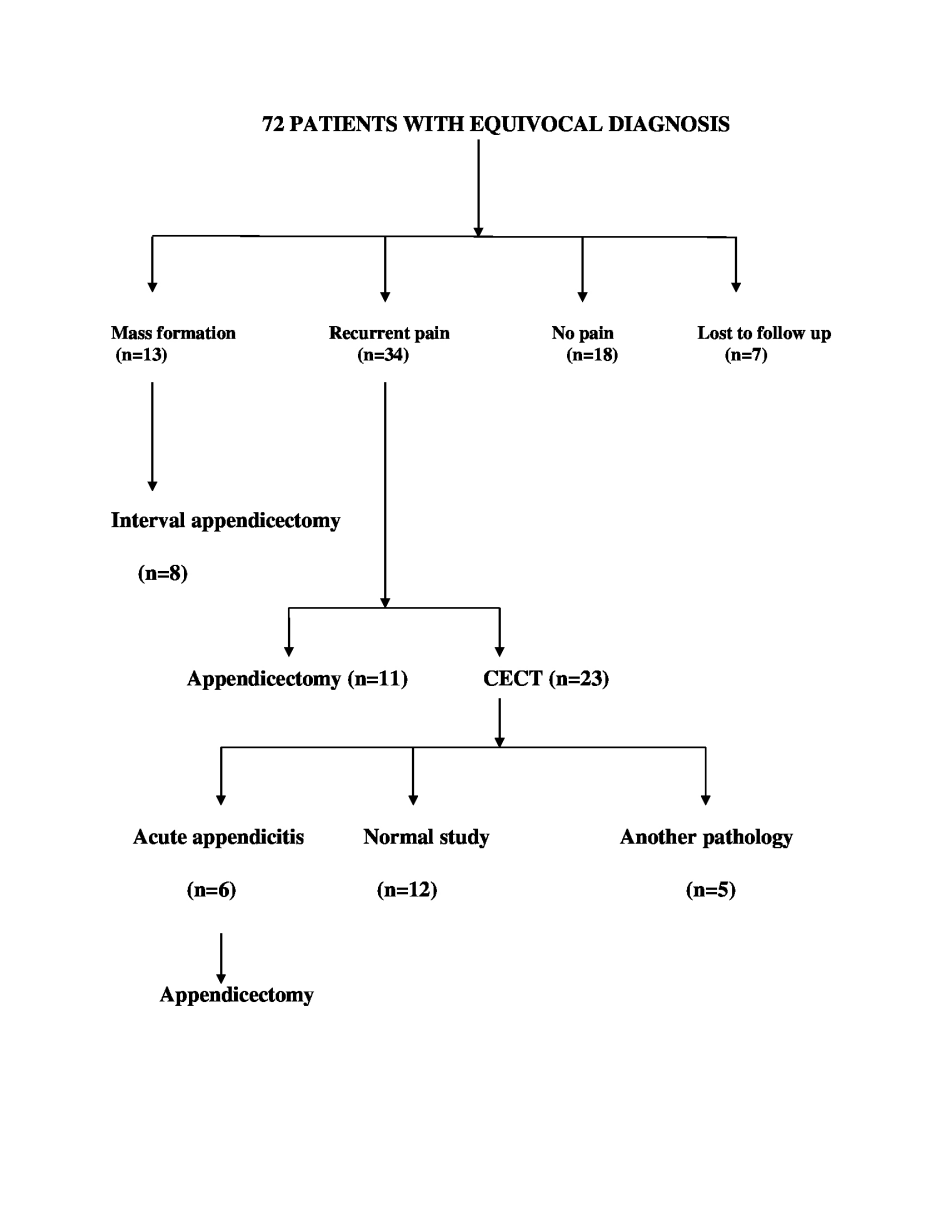
Scheme of outcome and management of patients with equivocal diagnosis CECT - contrast-enhanced computed tomography.

Among the 72 patients with equivocal diagnosis, 13 patients subsequently had RIF mass formation and were planned for interval appendicectomy. Eight patients followed up with interval appendicectomy and five were lost to follow-up. All eight patients who underwent interval appendicectomy had features of appendicitis on histology. During subsequent follow-up 34 out of 72 patients presented with recurrent pain, 11 patients underwent emergency appendicectomy, and CECT was done in the remaining 23 patients who had an equivocal diagnosis even on subsequent presentation. CECT showed features of appendicitis in six patients who subsequently underwent appendicectomy. CECT showed non-appendicular pathology in five patients. Another 18 out of the 72 patients had only a single self-limiting episode of RIF pain and were asymptomatic on follow-up. Out of the 72 patients, seven patients could not be followed up after their first admission. All 12 patients with a normal CECT study were symptomatically better on follow-up and none of them warranted admission or surgical intervention during the study period (Figure 1).

A total of 160 patients underwent appendicectomy. Based on the final histopathology report, these 160 patients were divided into normal appendix (n=14), uncomplicated appendix (n=37), and complicated appendix (n=109).

**Figure 2:**
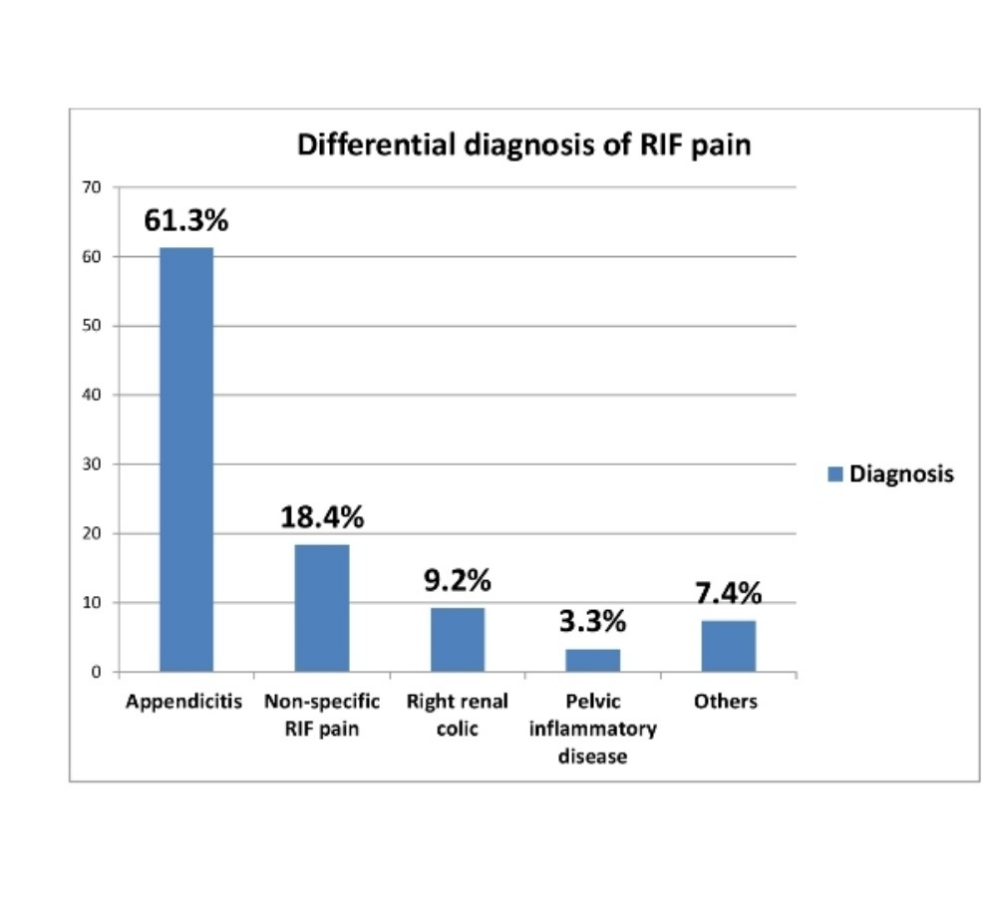
Differential diagnosis of RIF pain RIF - right iliac fossa.

The different aetiologies of RIF pain and the presenting symptoms/signs were analysed in the differential diagnosis of RIF pain (Table 1) (Figure 2).

**Table 1:** Clinical evaluation of RIF pain patients RIF - right iliac fossa.

Clinical symptoms & signs		Appendicitis(n=146)	Non-Appendicitis(n=92)	p value
Migrating pain	Yes	88(60.3%)	11(12%)	<0.05
	No	58(39.7%)	81(88%)	
Fever	Yes	104(71.2%)	46(50%)	<0.05
	No	42(28.8%)	46(50%)	
Anorexia	Yes	86(58.9%)	30(32.6%)	<0.05
	No	60(41.1%)	62(67.4%)	
Nausea/vomiting	Yes	103(70.5%)	43(46.7%)	>0.05
	No	43(29.45%)	48(53.3%)	
RIF guarding	Yes	85(58.2%)	13(14.1%)	<0.05
	No	61(41.8%)	79(85.9%)	
Rebound tenderness	Yes	105(71.9%)	16(17.4%)	<0.05
	No	41(28.1%)	76(82.6%)	

The mean values of total bilirubin (1.37 vs 0.89; p<0.05), direct bilirubin (0.44 vs 0.21; p<0.05), and total WBC counts (9574.2 vs 7880.9; p<0.05) were significantly higher in the appendicitis group when compared to the non-appendicitis group (Table 2).

**Table 2:** Laboratory parameters of RIF pain patients RIF - right iliac fossa.

Parameter		N	Mean	Standard deviation	p-value
Total count	Appendicitis	144	9574.2/mm^3^	2422.3	<0.05
	Non-appendicitis	84	7880.9/mm^3^	1865.2	
Total Bilirubin	Appendicitis	146	1.37mg/dl	0.53	<0.05
	Non-appendicitis	92	0.89mg/dl	0.36	
Direct Bilirubin	Appendicitis	146	0.44mg/dl	0.27	<0.05
	Non-appendicitis	92	0.21mg/dl	0.21	

Positive CRP levels (>0.6 mg/dl), USG diagnosing appendicitis, total WBC counts (>10,000), differential neutrophil counts (N>75%), an Alvarado score (> 4) between the groups were statically significant (p>0.05). CRP could not be done in 52 patients and total and differential counts could not be done in 10 patients (Table 3).

**Table 3:** Diagnostic value of laboratory investigations in RIF pain patients RIF - right iliac fossa, WBC - white blood cell, CRP - C-reactive protein, USG - ultrasonography.

Parameter	Appendicitis	Non-appendicitis	Total Number	p- value
WBC > 10,000/mm^3^	67(46.5%)	12(14.3%)	228	<0.05
WBC <10,000/ mm^3^	77(53.5%)	72(85.7%)		
Total	144	84		
Neutrophils>75%	94(65.3%)	22(26.2%)	228	<0.05
Neutrophils< 75%	50(34.7%)	62(73.8%)		
Total	144	84		
Bilirubin> 1.0mg/Dl	101(69.2%)	23(25%)	238	<0.05
Bilirubin< 1.0mg/Dl	45(30.8%)	69(75%)		
Total	146	92		
CRP positive	55(51.9%)	12(15%)	186	<0.05
CRP negative	51(48.1%)	68(85%)		
Total	106	80		
USG – Appendicitis	123(84.2%)	21(22.8%)	238	<0.05
USG –Non-appendicitis	23(15.8%)	71(77.2%)		
Total	146	92		
Alvarado >4	130(76.9%)	14(23.7%)	228	<0.05
Alvarado<4	39(23.1%)	45(76.3%)		
Total	169	59		

The total count had a low sensitivity and NPV (46.5% and 48.3%) but better specificity and PPV (85.7% and 84.8%). When other investigations like bilirubin, CRP, and USG were added on to total counts, it further increased the specificity and PPV but the sensitivity and NPV decreased significantly. USG as a single modality had a sensitivity of 84%, but when it was combined with total counts and serum bilirubin, sensitivity decreased to 40.3% and 58.9%, respectively. USG had a low specificity and PPV (77.2% and 85.4%), which could be increased by combining total counts (96.4% and 95.08%) and serum bilirubin (94.5% and 94.5%). The sensitivity and specificity of bilirubin value were 69.2% and 75%, respectively, and were higher than that of total WBC counts. Overall Alvarado scores had the maximum sensitivity (90.3%) and WBC counts had the maximum specificity (85.7%) for acute appendicitis when isolated investigations were considered. Combining two or more modalities increased the specificity further but at the cost of decrease in sensitivity (Table 4).

**Table 4:** Sensitivity, specificity, PPV, and NPV values of laboratory parameters of RIF pain patients PPV - positive predictive value, NPV - negative predictive value, WBC - white blood cell, CRP - C-reactive protein, USG - ultrasonography.

Parameter	Sensitivity (%)	Specificity (%)	PPV (%)	NPV (%)
WBC> 10,000 cells/mm^3^	46.5	85.7	84.8	48.3
Neutrophils >75%	65.3	73.8	81.0	55.3
Bilirubin> 1.0mg/dl	69.2	75	81.4	60.5
CRP positive	44.7	80.9	82.1	42.8
USG	84	77.2	85.4	75.5
Alvarado>4	90.3	53.6	76.9	76.3
WBC+ Bilirubin	37.5	95.2	93.1	47.05
WBC+CRP+ Bilirubin	27	97	92.8	52.7
WBC + USG	40.3	96.4	95.08	48.5
Bilirubin+ USG	58.9	94.5	94.5	59.2

History of migrating pain, vomiting, and presence of RIF guarding and tachycardia was seen more in complicated appendicitis compared with simple appendicitis (48.6% vs 19.3%; p <0.05). The presence of fever was not different between the two groups (Table 5).

**Table 5:** Comparison of signs and symptoms of simple vs complicated appendicitis RIF - right iliac fossa.

Symptoms/Signs	Simple appendicitis(n=109)	Perforated/ Gangrenous appendicitis(n=37)	p value
Migrating pain	58(53.2%)	30(81%)	<0.05
Fever	77(70.6%)	27(72.9%)	0.79
Vomiting	72(66%)	31(83.7%)	<0.05
Loss of appetite	65(59.6%)	21(56.7%)	0.76
RIF guarding	57(52.3%)	28(75.6%)	<0.05
Rebound tenderness	76(69.7%)	29(78.4%)	0.31
Pulse rate > 100	21(19.3%)	18(48.6%)	<0.05

The analysis of WBC counts (except differential counts), CRP, and serum bilirubin levels and their role as predictors in complicated appendicitis were not significant. The mean serum bilirubin level in the simple appendicitis and complicated appendicitis groups (1.28 mg/dl vs 1.61 mg/dl; p=0.02) was statistically significant. Among the patients with complicated appendicitis, CRP was positive in 69.5% of patients compared to 47% of patients with simple appendicitis. The sensitivity and specificity of CRP as predictors of perforation was 69% and 53%. The positive and negative predictive powers were 29.1% and 86.3%. Preoperative blood sample haemolysed in two patients and the CRP test kit was not available for 40 patients (Table 6).

**Table 6:** Analysis of laboratory tests in simple vs complicated appendicitis WBC - white blood cell, CRP - C- reactive protein.

Investigation	Simple Appendicitis	Complicated Appendicitis	Total Number	p Value
WBC >10,000/mm^3^	45(42%)	22(59.5%)	144	0.067
WBC<10,000/mm^3^	62(58%)	15(40.5%)		
Total	107	37		
Neutrophils>75%	68(63.5%)	31(83.8%)	144	0.022
Neutrophils<75%	39(36.5%)	6(16.2%)		
Total	107	37		
Bilirubin >1.0mg/dl	72(66.05%)	29(78.4%)	146	0.16
Bilirubin< 1.0mg/dl	37(33.95%)	8(21.6%)		
Total	109	37		
CRP positive	39(47%)	16(69.5%)	106	0.055
CRP negative	44(53%)	7(30.4%)		
Total	83	23		

The analysis of patients who underwent appendicectomy with postoperative histology showing no evidence of inflammation, showed that out of 14 patients, the WBC counts, serum bilirubin, and CRP were positive in 14.3%, 21.4%, and 15.3%, respectively. The mean value of bilirubin in groups based on the final histopathology report (0.87+0.29 vs 1.28+0.46 vs 1.62+0.65; p<0.05) was significant (Table 7).

**Table 7:** Analysis of patients with RIF pain with histopathology data RIF - right iliac fossa, WBC - white blood cell, CRP - C-reactive protein, USG - ultrasonography.

Parameter	Normal Appendix (n=14)	Appendicitis (n=146)	p-value
WBC >10,000/mm^3^	2(14.3%)	67(46.5%)	<0.05
WBC<10,000/mm^3^	12(85.7%)	77(53.5%)	
Total	14	144	
Neutrophils>75%	7(50%)	94(65.3%)	0.25
Neutrophils<75%	7(50%)	50(34.7%)	
Total	14	144	
Bilirubin >1.0mg/dl	3(21.4%)	101(69.2%)	<0.05
Bilirubin < 1.0mg/dl	11(78.5%)	45(30.8%)	
Total	14	146	
CRP positive	2(15.3%)	55(51.9%)	<0.05
CRP negative	11(84.7%)	51(48.1%)	
Total	13	106	
Alvarado>4	7(50%)	130(90.3%)	<0.05
Alvarado<4	7(50%)	14(9.7%)	
Total	14	144	
USG positive	9(64.3%)	123(84.2%)	0.072
USG negative	5(35.7%	23(15.8%)	
Total	14	146	

## Discussion

Though the majority of patients presenting to the hospital with RIF pain had a diagnosis of appendicitis, non-specific RIF pain was also a common cause of RIF pain. The male to female ratio among patients who presented with RIF pain in this study was 9:6.7 with a male preponderance. This was similar to the results of the study by Buckius et al. [4]. The rate of appendicular perforation ranged between 18.3 and 34.0% in different studies [5]. In the present study, a perforated/gangrenous appendix was found in 32.9% of males and in 20% of females with appendicitis.

The Alvarado score has been used commonly as a diagnostic tool for appendicitis, and Chan et al. have also suggested the Alvarado score as a screening method for admission as inpatients [6]. Its high sensitivity (90.3%) supported its value as a screening tool for probable appendicitis and admission. The utility of CRP in diagnosing appendicitis has been evaluated in many studies. Negative CRP levels would most likely be associated with normal appendix [7]. In contrary, Amalesh T et al. showed that the sensitivity, specificity, PPV, and NPV for CRP in appendicitis was 91%, 42%, 88%, and 48%, respectively, and that it may not be a useful tool to surgeons [8].

When CRP was taken alone, the positive predictive value was 94.7%, specificity was 72%, and sensitivity was 85.1% in a study done by Shefki Xharra et al. [9]. A CRP level more than 0.6 mg/dl would show agglutination and the test was considered positive. CRP was not found to be a useful indicator of appendicitis with a sensitivity and specificity of only 44% and 80%. CRP negativity was also not useful to rule out appendicitis in patients with RIF pain as the NPV was only 42%. The specificity, PPV, and NPV for Alvarado score (>4) were 53.6%, 76.9%, and 76.3%, respectively, which were comparable to another similar study [10].

Andrew Emmanuel et al. found that hyperbilirubinaemia had a high specificity of 88% and positive predictive value of 91% for simple acute appendicitis [11]. In this study, hyperbilirubinaemia (>1.0mg/dl) was seen in 69.2% of patients with appendicitis compared to only 25% of those without appendicitis. Among the patients with complicated appendicitis, 78.4% had hyperbilirubinaemia. The specificity of hyperbilirubinaemia as an indicator of appendicitis was 75% and the PPV was 81.4%. The sensitivity and specificity of serum bilirubin (>1.0mg/dl) as a predictor of complicated appendicitis were 78% and 33.9%, respectively. It was better compared to total WBC counts and CRP whose sensitivities were 59.5% and 69%, respectively.

The fairly better sensitivity and PPV with low specificity and NPV indicate that a positive USG favours diagnosis of acute appendicitis but a negative USG was not sufficient to rule out the diagnosis and discharge the patient. This was supported by a meta-analysis by Orr RK et al. in which they found that USG has a high false negative rate when used in patients with classical signs of appendicitis and high false positive rate in patients who are clinically having a low probability of appendicitis [12]. USG becomes the first modality of imaging investigation of choice in our country because of its high accuracy and lower cost.

In a single centre series, they found that CECT study preceded emergent appendectomy in 93.2% of patients in 2007 compared to only 18.5% of patients in 1998. This also coincided with a decrease in the negative appendectomy rate from 16.7% in 1998 to 8.7% in 2007 [13]. Despite the high sensitivity and specificity of CECT in the diagnosis of appendicitis, its role is limited due to its cost factors, availability, radiation hazards, and further delay in surgical intervention. Recurrent RIF pain with no definitive cause identified is a known entity involving significant number of patients as shown in several studies [14]. Modalities like CECT and diagnostic laparoscopy have been the next line of management for these patients but with variable results. In different studies non-specific RIF pain has been described [15]. In this study, 18.5% of patients belonged to this group.

In a similar study by Xharra S et al., they found that WBC count had a sensitivity of 79.1% and specificity of 68% for a cut off value of 10,000/mm3 [9]. In another retrospective study by Kim E et al., they found that for the same cut off value WBC had a sensitivity of 81% but poor specificity of only 22%, which is controversial with the results of our study, which showed a better specificity [16]. There was a significantly higher negative predictive value for WBC counts when all causes of RIF pain were included as negative samples. Whether elevated counts help predict complicated appendicitis has been evaluated in different studies.

The Alvarado score (>4) was seen in 50% of patients. Among the patients who were initially discharged as non-appendicitis and who on their subsequent presentations underwent appendicectomy, none of them had complicated appendicitis. This may be due to the early presentation as a result of better patient awareness created during their previous discharge from hospital, as many studies have shown a direct relationship of complicated appendicitis with duration after onset of pain.

The ultimate goal of the present study was to find out the ways to reduce the negative appendicectomy rates and unnecessary admissions for more benign causes of RIF pain. The estimated negative appendicectomies (8.2%) was lesser compared to different other studies where it ranged from 17% to 23%. It could significantly bring down the health care costs.

In comparison to other studies, the efficacy of biochemical parameters were compared with the non-appendix group in this study. This study group might not actually be a representative of the profile of all patients with RIF pain since some of the patients were partially investigated from other referring hospitals. The CRP levels could only be measured by the semi quantitative agglutination method due to the non-availability of an automated nephlometer. In some patients, CRP and WBC counts could not be carried out due to improper sampling/non availability of test kits.

## Conclusions

Amongst all the laboratory tests, serum bilirubin was found to have better sensitivity and negative predictive values than WBC counts and CRP in diagnosing acute appendicitis. USG of the abdomen had an important role in the diagnosis of appendicitis with significant sensitivity, specificity, and PPV. CECT was useful in the evaluation of recurrent undiagnosed RIF pain. Use of laboratory investigations and USG imaging as an adjunct to clinical diagnosis will help to diagnose acute appendicitis in patients with RIF pain.
